# Multi-Smart and Scalable Bioligands-Free Nanomedical Platform for Intratumorally Targeted Tambjamine Delivery, a Difficult to Administrate Highly Cytotoxic Drug

**DOI:** 10.3390/biomedicines9050508

**Published:** 2021-05-04

**Authors:** Marta Pérez-Hernández, Cristina Cuscó, Cristina Benítez-García, Joaquin Bonelli, Marina Nuevo-Fonoll, Aroa Soriano, David Martínez-García, Alain Arias-Betancur, María García-Valverde, Miguel F. Segura, Roberto Quesada, Josep Rocas, Vanessa Soto-Cerrato, Ricardo Pérez-Tomás

**Affiliations:** 1 Cancer Cell Biology Research Group (CCBRG), Department of Pathology and Experimental Therapeutics, Faculty of Medicine and Health Sciences, Universitat de Barcelona, L’Hospitalet de Llobregat, 08907 Barcelona, Spain; martaperezh@ub.edu (M.P.-H.); cristinabg1996@gmail.com (C.B.-G.); marina_nufo@hotmail.com (M.N.-F.); davidmg.ub@gmail.com (D.M.-G.); alain.arias@ub.edu (A.A.-B.); vsoto@ub.edu (V.S.-C.); 2 Oncobell Program, Institut d’Investigació Biomèdica de Bellvitge (IDIBELL), L’Hospitalet de Llobregat, 08908 Barcelona, Spain; 3 Nanobiotechnological Polymers Division, Ecopol Tech, S.L., El Foix Business Park, Indústria 7, 43720 L’Arboç del Penedès, Spain; criscusco65@gmail.com (C.C.); joaquin.bonelli@ecopoltech.com (J.B.); direccio@ecopoltech.com (J.R.); 4 Group of Translational Research in Child and Adolescent Cancer, Vall d’Hebron Research Institute, Universidad Autónoma de Barcelona, 08035 Barcelona, Spain; aroa.soriano@vhir.org (A.S.); miguel.segura@vhir.org (M.F.S.); 5 Department of Integral Adult Dentistry, Research Centre for Dental Sciences (CICO), Universidad de La Frontera, Temuco 4811230, Chile; 6 Department of Chemistry, Universidad de Burgos, 09001 Burgos, Spain; magaval@ubu.es (M.G.-V.); rquesada@ubu.es (R.Q.)

**Keywords:** polymer nanocapsules, tumor microenvironment, pH-tunable, lung cancer treatment, targeted drug delivery systems, amphoteric nanocapsules

## Abstract

Cancer is one of the leading causes of mortality worldwide due, in part, to limited success of some current therapeutic approaches. The clinical potential of many promising drugs is restricted by their systemic toxicity and lack of selectivity towards cancer cells, leading to insufficient drug concentration at the tumor site. To overcome these hurdles, we developed a novel drug delivery system based on polyurea/polyurethane nanocapsules (NCs) showing pH-synchronized amphoteric properties that facilitate their accumulation and selectivity into acidic tissues, such as tumor microenvironment. We have demonstrated that the anticancer drug used in this study, a hydrophobic anionophore named T21, increases its cytotoxic activity in acidic conditions when nanoencapsulated, which correlates with a more efficient cellular internalization. A biodistribution assay performed in mice has shown that the NCs are able to reach the tumor and the observed systemic toxicity of the free drug is significantly reduced in vivo when nanoencapsulated. Additionally, T21 antitumor activity is preserved, accompanied by tumor mass reduction compared to control mice. Altogether, this work shows these NCs as a potential drug delivery system able to reach the tumor microenvironment, reducing the undesired systemic toxic effects. Moreover, these nanosystems are prepared under scalable methodologies and straightforward process, and provide tumor selectivity through a smart mechanism independent of targeting ligands.

## 1. Introduction

Conventional chemotherapy cannot act specifically on cancer cells in a targeted manner, which results in damage to healthy tissues and considerable secondary adverse effects. This is why new therapeutic approaches are focused on targeted therapies and novel tumor-targeted delivery systems.

Nanomedicine, the application of nanotechnology in medicine and healthcare, is understood to be a key enabling instrument for personalized medicine by delivering next generation therapies. In this sense, nanomedicines have the potential to overcome current limitations of conventional therapies providing, among others, selectivity to target tissues, controlled drug release and protection against premature inactivation [1]. Since nanoparticles show high surface-to-volume ratios, the surface properties have a tremendous impact on the final nanosystem performance. According to this, adjusting surface and the physicochemical properties of the nanoparticles results in differentiated behaviors in vitro and in vivo. They can be modulated in terms of size, material, surface charge, nature, and biofunctionalization, among others. Concerning size, small nanoparticles generally show an enhanced intratumoral penetration [2,3]; however, they can be rapidly cleared from the circulation before reaching the tumor site. In regard to the surface properties, hydrophilic coating polymers such as polyethylene glycol [4] can prolong blood circulation and decrease immunogenicity, which improves their distribution and accumulation into tumors through the Enhanced Permeability and Retention (EPR) effect [5,6]. Regarding surface charge, anionic untargeted nanoparticles are usually poorly internalized, whereas positively charged ones present strong interaction with cell membranes, stimulating their cellular uptake [7,8]. However, such a cellular uptake does not occur exclusively in tumor cells, since both tumor and healthy cells can equally internalize cationic nanoparticles. According to this, in order to differentiate between healthy and tumor cells, an ideal system should have an optimal nanoparticle size and show anionic to neutral properties in normal tissues—to avoid internalization—and positively charged surface in tumor conditions, to increase cell penetration.

The tumor microenvironment is characterized by a slightly acidic extracellular pH (pHe 6.3–6.9), compared to a normal tissue (pHe 7.4), due to a change in the metabolism of tumor cells to an aerobic glycolysis, known as the Warburg effect [9]. This reversed pH provides cells with adaptive advantages that promote cancer progression such as high proliferation rate, evasion of apoptosis, and metastatic characteristics [10,11]. Several researches have reported that differences in pH between tumor and healthy tissue can be exploited to promote targeted anticancer therapy [12]. This has led to the exploration of different nanosized systems with pH-responsive properties for cancer treatment [13]. One example is the development of nanoparticles with a large structure to avoid blood clearance that shrink once pH decreases in tumor, enhancing cell penetration [14]. Another study describes the synthesis of nanosystems that are chemically stable under physiological conditions but release the encapsulated drug when pH conditions become acidic, showing an improved tolerability in blood [15].

In this paper, we focused on polyurea/polyurethane NCs due to the wide range of possibilities offered by these materials. They have been reported as one of the most versatile products used for different biomedical purposes. In fact, they can be prepared and easily customized to meet the desired properties, since the reactions are efficient, clean, and allow multiple starting products. Moreover, due to the nature of polyurea and polyurethanes, and the industrial availability of reactants, the methodologies can be scaled and the nanoparticles can be obtained in pilot or production plants [16].

More particularly, we describe the nanoencapsulation of an experimental cytotoxic drug named tambjamine 21 (T21) [17,18] into a multi-sensitive drug delivery system that is highly sensitive to small changes on pH conditions (WO2014114838A2) [19,20,21,22,23]. When pH conditions become acidic as a result of the tumor microenvironment, nanoparticles turn into cationic entities, boosting their cell uptake. Besides, these nanosystems show a good loading capacity for lipophilic drugs, that can be easily modulated depending on the hydrophobic core and are selectively degraded through a redox-triggered process involving reduced GSH [24].

The cytotoxic cellular effects of the NCs on tumor and non-tumor cell lines and their cellular internalization are studied in different pH conditions, as well as biodistribution, safety profile, and therapeutic activity in vivo.

## 2. Materials and Methods

### 2.1. Tambjamine 21 Synthesis

Tambjamine analogue number 21 (T21) was synthesized as previously reported [17]. The drug was dissolved at 10 mM (3.6 mg/mL) in DMSO (dimethyl sulfoxide) and stored at −20 °C. Subsequent dilutions for biological assays were made in phosphate-buffered saline (PBS) or media with a maximum concentration of 1% DMSO for in vitro assays and in PBS with 7.5% DMSO and 0.8% Tween-20 for in vivo studies.

### 2.2. Synthesis of Nanocapsules

All variants of NCs were synthesized by using a modification of the synthetic approach developed by ECOPOL TECH [22,24]. Different types of polyurea/polyurethane polymers were synthesized through a two-step process consisting of polyaddition reactions between nucleophilic monomers (alcohols and amines) and isophorone diisocyanate. The resulting polymer was subsequently used to efficiently nanoencapsulate the target lipophilic molecules under mild and aqueous conditions. Finally, once the NCs were prepared, the nanodispersion was purified by dialysis to remove non-reacted and non-encapsulated products and was fully characterized. All the synthetic procedures are described in detail in the SI (Figures S1–S4 and Tables S1–S6).

### 2.3. Cell Culture

A549 (human lung adenocarcinoma) and LLC1 (mouse Lewis lung carcinoma) cells were cultured with DMEM high glucose from Biological Industries (Beit Haemek, Israel), 10% FBS (Fetal bovine serum) from Gibco (ThermoFisher Scientific, Waltham, MA, USA). In some of the experiments another media was also used, which differs in the quantity of NaHCO_3_ leading to a different pH (Figure S5). Cells were cultured in these two media, referred hereafter as pH 7.8 and pH 6.8, respectively. MCF10A cells (non-tumorigenic human cell line) were cultured with DMEM/F12 1:1 medium, 5% HS (Horse serum), 100 ng/mL cholera toxin (Calbiochem, San Diego, CA, USA), 10 µg/mL human insulin, 10 µg/mL epidermal growth factor and 0.5 µg/mL hydrocortisone (all from Sigma-Aldrich Chemical Co., Saint Louis, MO, USA). The CHLA-90 cell line was obtained from the Children’s Oncology Group cell line repository. CHLA-90 cells were cultured and maintained in Iscove’s Modified Dulbecco’s Medium (Life Technologies, Thermo Fisher Scientific, Waltham, MA, USA), supplemented with 20% heat-inactivated FBS South America Premium (Biowest, Nuaillé, France), 1% Insulin-Transferrin-Selenium G Supplement (Life Technologies, Thermo Fisher Scientific, Waltham, MA, USA), 100 U/mL penicillin, 100 μg/mL streptomycin (Life Technologies, Thermo Fisher Scientific, Waltham, MA, USA) and 5 μg/mL of Plasmocin Prophylactic (InvivoGen, San Diego, CA, USA).

### 2.4. Nanocapsules Internalization by Confocal Microscopy

The cells were seeded at concentration of 10^5^ cells/mL in a 12-well plate containing glass coverslips (18 mm diameter) pretreated with 0.01% polylysine (Sigma-Aldrich, Saint Louis, MO, USA) and allowed to attach for 24 h. Then, cells were incubated with 10 µg/mL DiO encapsulated amphoteric nanocapsules (NC-DiO) at pH 7.8 for 1–48 h. The cells were then washed twice with PBS, incubated with 0.25 µM LysoTracker Red (DND-99) (Molecular Probes, Eugene, OR, USA) for 1 h, and fixed with 4% paraformaldehyde (PFA) for 20 min at 4 °C. Nuclei were dyed with Hoechst 33342 (Sigma-Aldrich, Saint Louis, MO, USA) and slides were mounted on Mowiol (Sigma-Aldrich, Saint Louis, MO, USA). The immunofluorescence images were captured using a Leica fluorescent microscope (Leica Microsystems, Wetzlar, Germany).

### 2.5. Cell Viability Assay (MTT)

5 × 10^3^ A549 cells and 10^4^ LLC1 and MCF10A cells were seeded in 80 µL of medium in 96-well plate and incubated overnight at 37 °C, 5% CO_2_. Nanocapsules and free T21 were added at different concentrations in 20 µL and incubated for 24, 48 and 72 h. MTT (3-(4,5dimethylthiazol-2-yl)-2,5 diphenyltetrazolium bromide) was added at 0.5 mg/mL final concentration and incubated for 2 h at 37 °C, 5% CO_2_. Then, the supernatant was removed, formazan crystals were dissolved in 100 µL of DMSO (Sigma-Aldrich, Saint Louis, MO, USA), and absorbance was measured at 570 nm using the plate reader Multiskan FC (Thermo Scientific, Waltham, MA, USA).

### 2.6. Nanocapsules Internalization by Flow Cytometry

A549 cells were seeded at 10^5^ cells/mL in 24-well plate (total volume 1 mL) at pH 7.8 and 6.8 and incubated overnight at 37 °C, 5% CO_2_. Amphoteric or anionic nanocapsules loaded with DiO (NC-DiO) were added (180 µg/mL shell) and incubated for 1, 15, 24, 48 and 72 h. Cells were washed with PBS, trypsinized and analyzed by flow cytometry using the cytometer BD FACSCanto II (BD Bioscience, San Jose, CA, USA).

### 2.7. Nanocapsules Biodistribution Assay

All animal procedures were approved by the Ethics Committee for Animal Experimentation of Vall Hebron Research Institute (ref-52/18 CEEA). CHLA-90 cells (5 × 10^6^) were injected into the right flank of 6- to 8-week-old female NMRI-nude mice (Janvier Labs, Le Genest-Saint-Isle, France) in 300 μL of PBS:Matrigel (1:1) (Corning, New York, NY, USA). Tumor volume was measured every 2–3 days. Once the tumors reached a median volume of ~250 mm^3^, the mice were randomized in two groups (NC-DiR and NC-DiR-T21). The mice (*n* = 5/group) were treated with a unique dose of nanoparticles loaded with fluorophore DiR (NC-DiR) or fluorophore DiR and T21 (NC-DiR-T21) at 0.4 mg DiR/kg by intravenous (i.v.) administration. For each group, one mouse was injected with PBS to be used as a negative control of tissue auto-fluorescence. At 72 h post injection, the animals were euthanized; liver, spleen, kidneys, lungs, and tumor were collected. Nanoparticle tissue accumulations were determined by ex vivo DiR fluorescence imaging using the IVIS^®^ Spectrum imaging system. Images and measurements of fluorescent signals were acquired and analyzed using Living Image^®^ 4.5 software (PerkinElmer, Waltham, MA, USA). The fluorescence signal was quantified in radiant efficiency units (REU); the fluorescence emission radiance per incident excitation power. The area of each tissue was determined to calculate the total REU/mm^2^. Fluorescent signal was normalized versus the total REU/mm^2^ of PBS-treated mice. Results are shown as percentage of total detected fluorescence in the analyzed organs.

### 2.8. In Vivo Toxicity Study

C57BL6/FVBN/B6SJL 6- to 7-months-old mice were divided into 6 groups of 3 mice per group and administered intravenously (i.v.) with 6 mg/kg or 3 mg/kg of NC-T21 in PBS. The control groups were also administered i.v. with PBS (V) or with empty nanocapsules without T21 (NC). Another group was treated with free T21 administered intraperitoneally (i.p.) at a concentration of 6 mg/kg, and its own control with the solvent used; PBS 7.5% DMSO and 0.8% Tween-20 (Vip). 72 h after the last dose, the mice were sacrificed, and samples were collected and analyzed, as described below.

### 2.9. Orthotopic Lung Cancer Model

All animal studies were approved by the Autonomic Ethic Committee (Generalitat de Catalunya) under the protocol 9394. Adenovirus expressing the Cre-recombinase were administered intranasally to trigger the expression of cre-inducible *Kras* mutation, which initiates the development of lung tumors [25]. Virus solution was prepared adding 5 × 10^7^ pfu/mouse of virus provided by the Viral Vector Production Unit from Universitat Autònoma de Barcelona, (Prep# UPV-757 23/10/2014) to EMEM medium (Biological Industries) with 12 mM CaCl_2_ final concentration and incubated for 20 min at room temperature for virus precipitation. Mice with an oncogenic mutation in *Kras* (gly → asp codon 12) flanked by LoxP sites were anesthetized with 80 mg/kg ketamine (Ketolar^®^ from Pfizer, Madrid, Spain) and 20 mg/kg xylacine (Xilagesia; from Calier, Barcelona, Spain) and administered via intranasal with 30 µL of the adenovirus solution. The transient expression of Cre allows the expression of the oncogenic mutation and consequently the development of lung tumors in 13 weeks. Mice were divided into 4 groups of 8 mice per group and 13 weeks after virus inhalation, the treatment started. Mice were treated twice a week with 6 mg/kg of nanoencapsulated T21 in PBS i.v. (NC-T21); with PBS i.v. (V) or with empty nanocapsules without T21 i.v. (NC). The last group was treated with 6 mg/kg of free T21 i.p. (T21).

Mice weight was monitored weekly since the virus administration started and every two days during treatment. Mice were sacrificed after 10 doses (60 mg/kg of the drug); the organs were collected and analyzed.

### 2.10. Mice Sample Collection and Processing

Mice were sacrificed in a CO_2_ chamber and the principal organs were collected, weighed, and processed as detailed bellow.

#### 2.10.1. Tissue Processing in Paraffin

The samples were processed in a tissue processor Shandun Citadel 1000 (Thermo Scientific, Waltham, MA, USA) as follows; two washing steps in PBS pH 7.4 for 2 h and 1 h 30 min, following by gradual dehydration in solutions with increasing concentrations of ethanol. 1 h 30 min at 30%, 2 h at 70%, 2 h at 96% and twice in absolute ethanol for 2 h each. Then, the samples were cleared twice in xylene for 1 h 30 min and 2 h and infiltrated in paraffin wax (PanReac Applichem, Castellar del Vallès, Spain) overnight. Tissue was embedded in blocks of paraffin and left to chill in the fridge minimum 1 h and stored at room temperature. Afterwards the excess of paraffin was removed, blocks were precooled 1 h at 4 °C and sections of 4 µm were cut using a microtome Jung Biocut 2035 (Leica Microsystems) and fixed in microscope slides coated with polylysine.

#### 2.10.2. Hematoxylin-Eosin Staining from Paraffin Preparations

The slides were placed in heating oven for 30 min at 60 °C in order to melt the paraffin and were deparaffinized in xylene twice for 3 min each. After gradual hydration in solutions for 2 min each, with decreasing concentration of ethanol (100%, 96%, 80%, 70%) and distilled H_2_O (dH_2_O), the slides were stained with Harris hematoxylin for 3 min 30 s and washed with tap H_2_O, changing the water until it appears clean; then the unspecific stain was removed with hydrochloric acid-alcohol (70% ethanol, 0.35% hydrochloric acid) for 1 s and washed with tap H_2_O for 3 s, 0.03% ammonia H_2_O for 3 s, dH_2_O for 5 min, 70% ethanol for 3 s, 80% ethanol for 3 s and stained with eosin for 1 min and 30 s. Then, the slides were washed with dH_2_O twice so as to clean them thoroughly and dehydrated with increasing concentrations of ethanol (96% twice for 3 s, absolute ethanol for 3 s and 3 min), ethanol-xylene 1:1 for 5 min, xylene 3 times of 3 min with a drop of eucalyptol in the last one. Finally, the coverslips were mounted on the slides with DPX.

## 3. Results and Discussion

Firstly, we focused on the design of a multi-sensitive drug delivery system that could lead to a preferential uptake of the chemotherapeutic drugs by tumor cells, an increase of drug tolerability, and a minimization of undesired cytotoxic side effects. To this end, we encapsulated the antitumor drug T21 into amphoteric GSH-sensitive NCs, taking advantage of the pH shift that occurs between healthy cells and the tumor microenvironment. In this sense, the objective was to prepare a biocompatible nanosystem with the ability to respond to this pH change, becoming a highly penetrating entity in tumor conditions (slightly acidic pH) and remaining mainly inert in physiological state.

Secondly, we characterized the NCs, studied their cell uptake and cytotoxic effect depending on pH (in vitro) and we analyzed their biodistribution, safety profile, and efficacy (in vivo).

### 3.1. Nanocapsules Synthesis and Characterization

**Scheme 1:**
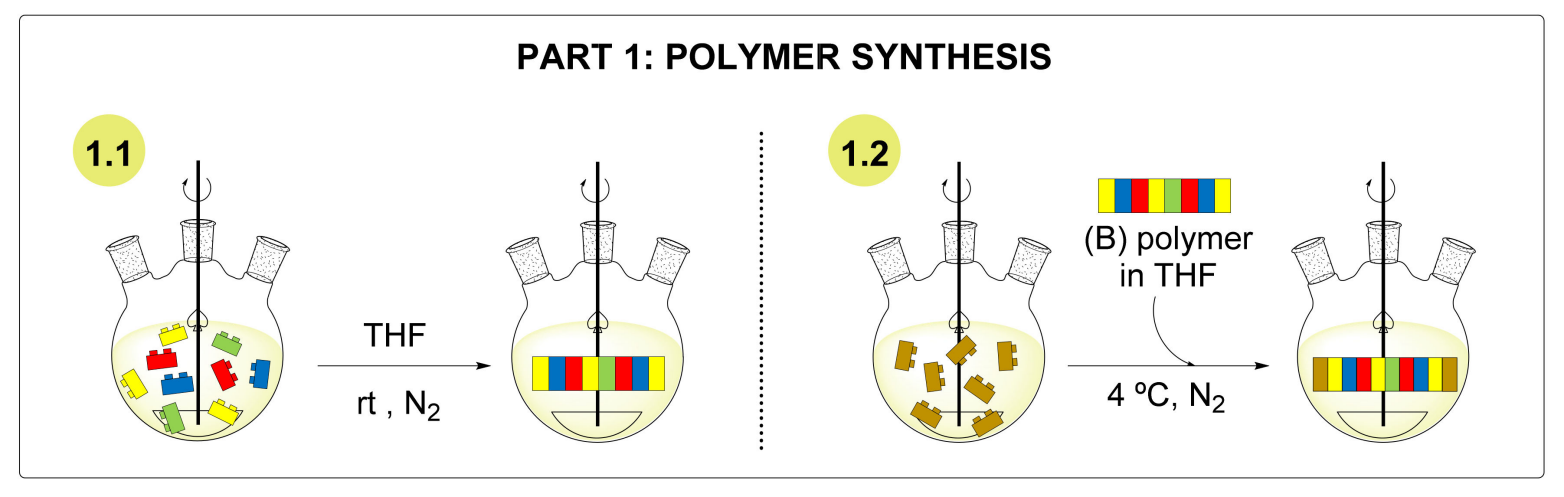
Steps involved in the preparation of the polymer. THF: tetrahydrofuran. rt: room temperature.

The synthesis of nanocapsules started with the preparation of the polymer. This preparation was split into two different steps, as shown in Scheme 1 [22,24].

The first part of the polyaddition reaction (1.1 of the scheme) involved the polymerization between the diisocyanate and the diols, resulting in an NCO-reactive polymer containing new urethane bonds. In the next step (1.2 of the scheme), the polymer was end-capped by the diamine, leaving free amine residues to allow reactivation in the nanoencapsulation step.

**Scheme 2:**
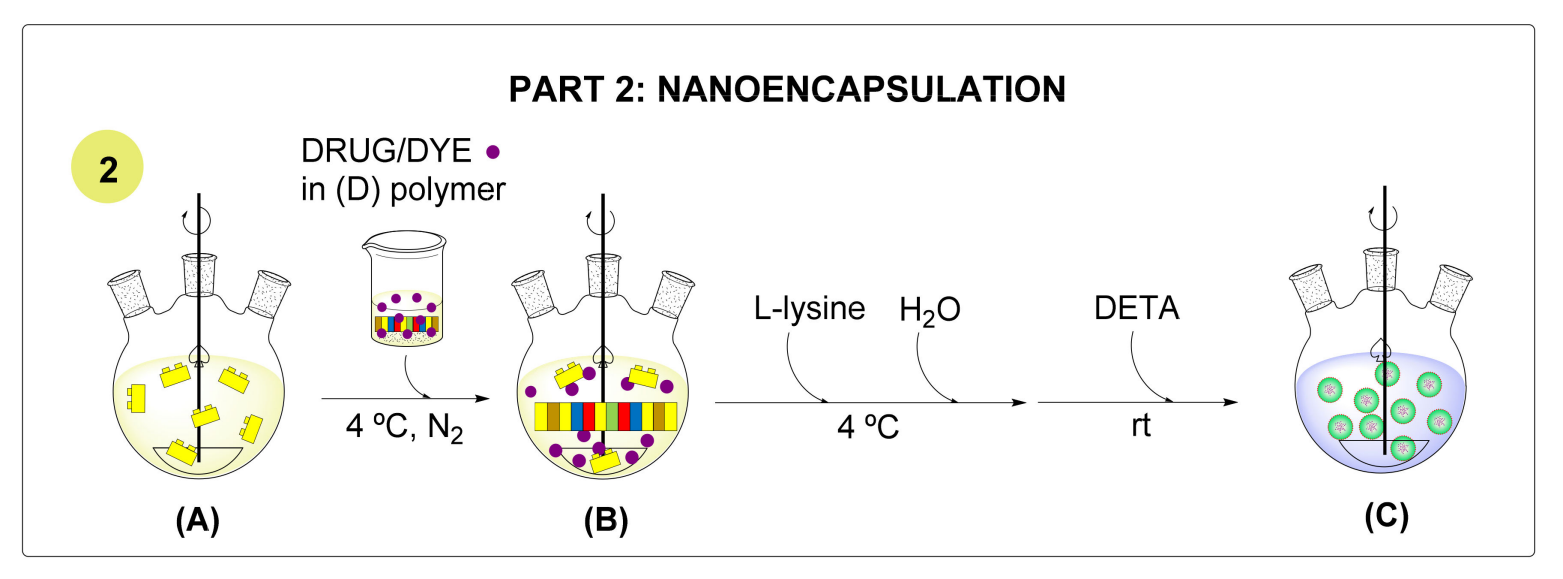
Graphic representation of the nanoencapsulation process.

The antitumor drug T21 and two different lipophilic fluorophores DiO (3,3′-Dioctadecyloxacarbocyanine Perchlorate) and DiR (DiIC18(7); 1,1′-dioctadecyl-3,3,3′,3′-tetramethylindotricarbocyanine iodide) were selected to prepare different NC analogues. The general nanoencapsulation process is detailed in Scheme 2.

The process started with the addition of a mixture of the polymer and the target fluorophore/drug cargo to a solution of the diisocyanate (A, B). Once the polymer reacted with the NCO groups, it was reactivated, and l-lysine sodium salt was introduced as an ionomer. Finally, water was added dropwise to turn the organic phase into an o/w nanoemulsion and the process finalized with the addition of a triamine, which rapidly crosslinked the NCO-reactive polymers gathered in the interface to generate the desired NCs (C). The NCs were purified by dialysis, lyophilized, and fully characterized. Following these procedures, NCs encapsulating the three target molecules (NC-DiR, NC-DiO, NC-T21) as well as NCs encapsulating both fluorophore DiR and T21 (NC-DiR-T21) were produced. Two reference NCs were also prepared as a control on the amphoteric properties (NC-DiO-AN) and as unloaded NCs (NC). Subsequently, the morphology, size, surface properties, encapsulation efficiency, and drug loading were determined using different characterization techniques (see SI for details).

**Figure 1:**
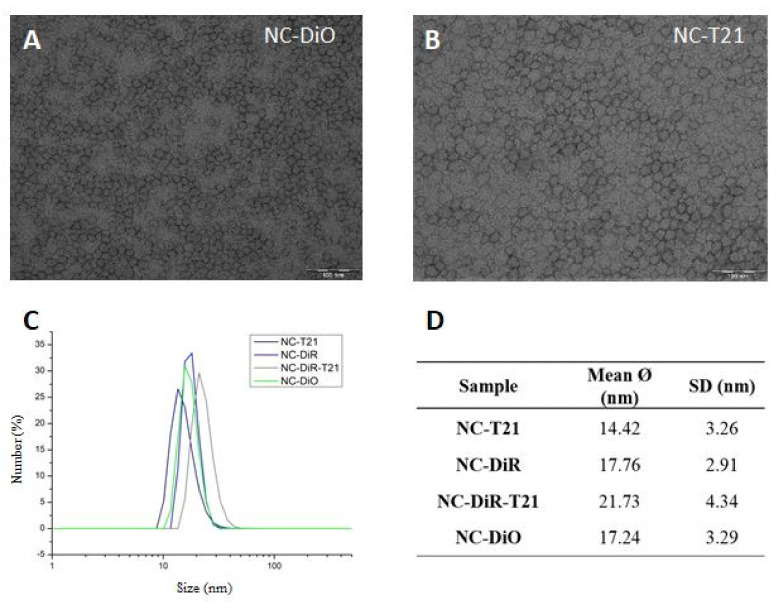
Characterization of the nanocapsules. (**A**,**B**) representative TEM micrographs (scale bar: 100 nm); (**C**) particle size distributions by DLS; (**D**) statistical analysis of different NCs.

The NCs were characterized by transmission electron microscopy (TEM) and dynamic light scattering (DLS) in order to study their morphology and size, respectively (Figure 1).

According to Figure 1A,B TEM micrographs of the samples analyzed (NC-DiO and NC-T21, respectively) show the nanocapsules with a roughly homogeneous round shape. According to Figure 1C,D, the particle size distributions show monodispersity among all the samples analyzed, with an average particle size between 14 and 21 nm, with low SD values.

**Figure 2:**
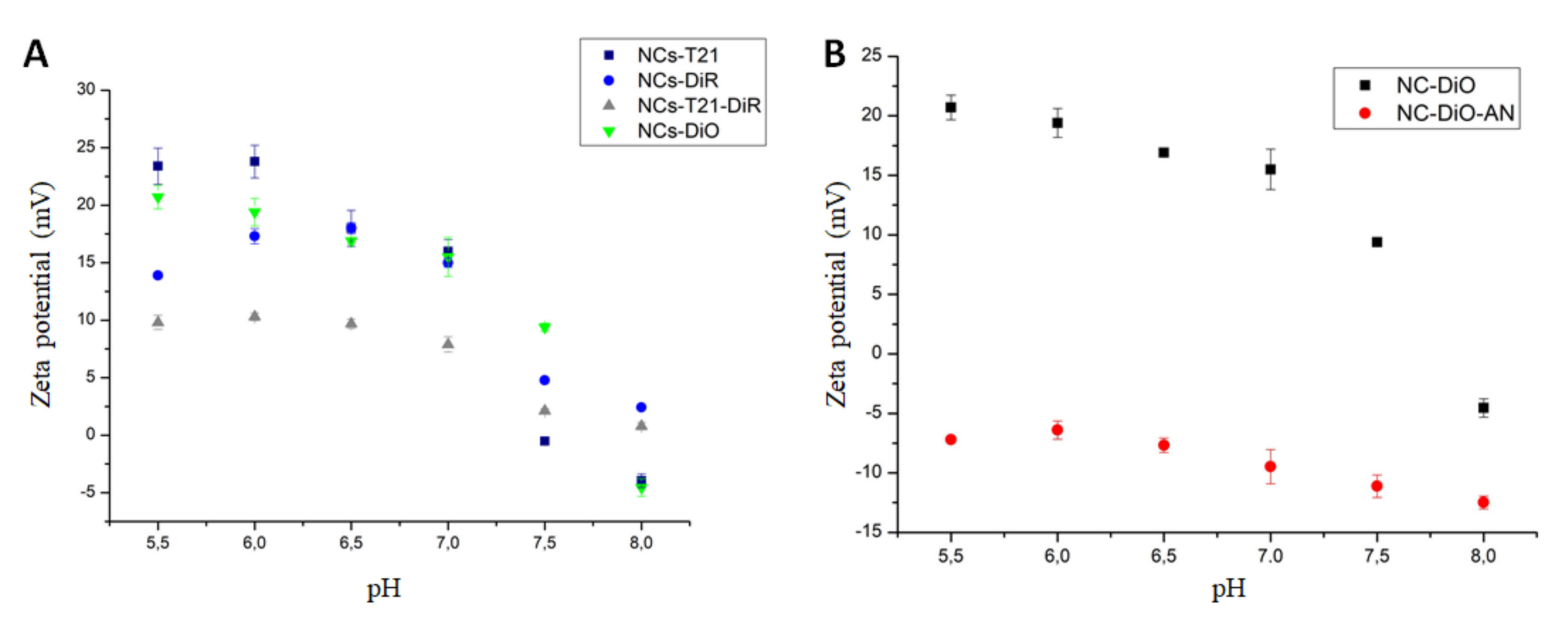
Zeta potential measurements. (**A**) Zeta potential values of 4 samples of NCs and (**B**) comparative analysis to a control NCs (NC-DiO-AN).

The surface properties of the whole range of NCs synthesized were studied by zeta potential (Figure 2) in different pH conditions, from pH 5.5 to pH 8.5 in order to see the differences on the surface charge in a relevant pH range (mimicking physiological conditions and the tumor microenvironment).

According to the results shown in Figure 2A, all the NCs showed a cationic tendency when the pH of the medium turned acidic and their zeta potential dropped considerably in physiological to slightly anionic conditions. These observations can be explained by the selective protonation of the Jeffcat DPA amine groups and its synchronized effect with lysine sodium salt [24]. Interestingly, this effect is more pronounced for the NC encapsulating T21. This result can be explained because T21 is a cationic compound which can be partially deprotonated at slightly basic pH, contributing to the observed change in zeta potential. The amphoteric nature and pH-responsiveness properties of the selected polymers used to nanoencapsulate the molecules were further supported by the results obtained when compared with non-amphoteric NCs (NC-DiO-AN) (Figure 2B). In the same pH conditions, these NCs show invariable neutral to anionic surfaces, regardless of the pH conditions and the tendency showed by NC-DiO towards more positive zeta potential at acidic pHs is lost.

### 3.2. NCs Internalization into Cells

**Figure 3:**
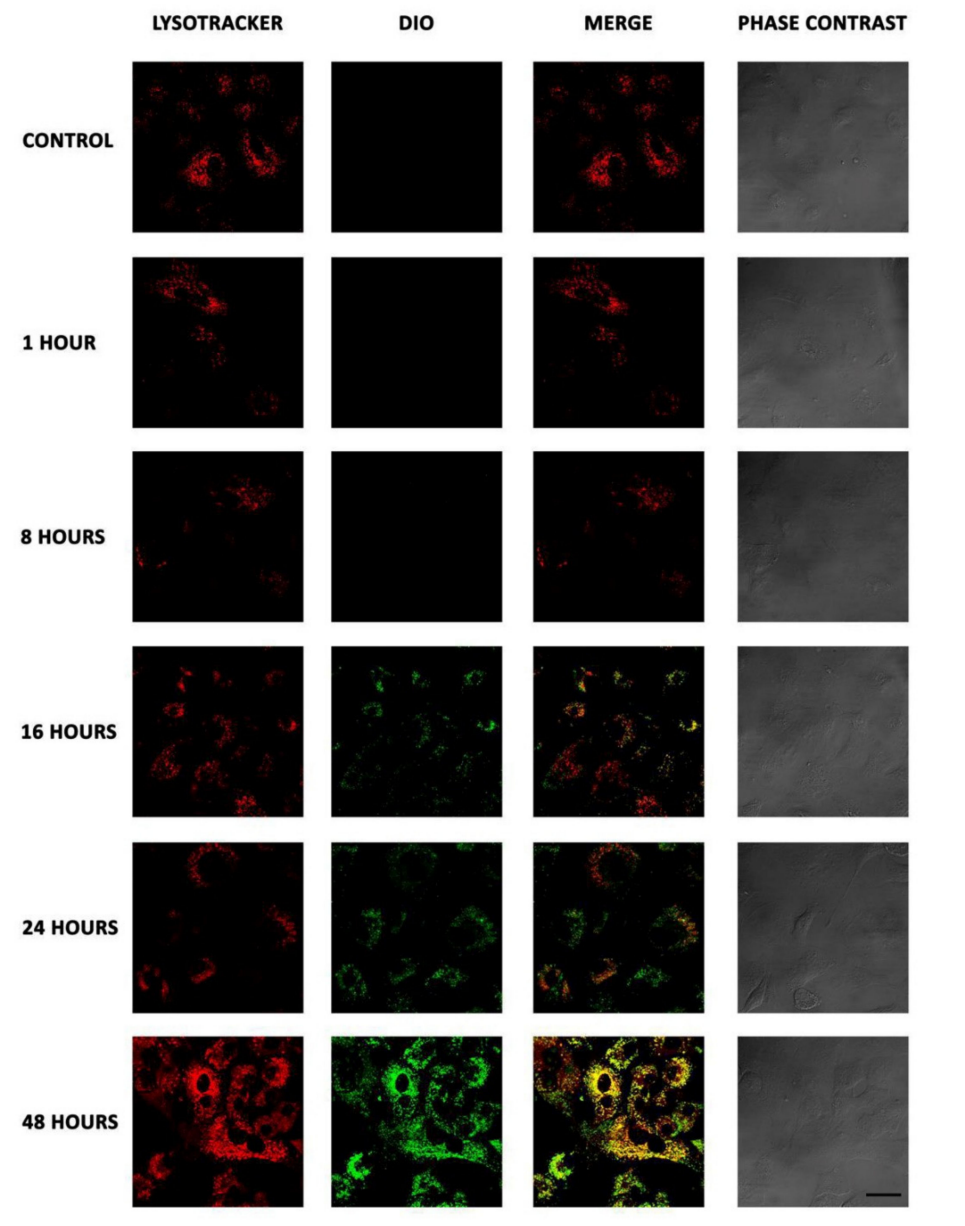
Internalization of NCs by confocal microscopy. A549 cells were incubated with NC-DiO (green) for 1, 8, 16, 24, and 48 h, fixed and incubated with LysoTracker (red) to dye the late endosomes and lysosomes. Merge images show colocalization between NCs and acidic organelles (yellow). Scale bar 30 µm.

To assess NCs cellular internalization, NCs uptake was evaluated in A549 cells incubated during different time points (1 to 48 h) by confocal microscopy (Figure 3).

NCs were internalized into the cells in a time-dependent manner, being detected at 16 h and showing the highest levels of internalization at 48 h. On the other hand, merge images showed colocalization of the NCs into acidic organelles, such as late endosomes or lysosomes, which suggests that NCs are endocytosed, ending in lysosomes as most of the nanoparticles. This is considered a limitation for the use of nanoparticles as drug delivery systems [26]. The fact that these NCs may end in degradative organelles could limit their efficacy as drug carriers impeding the drug to be delivered into the cytosol for different cellular clearing mechanisms such as autophagy or exocytosis. However, our experimental drug (T21) can cross lipid membranes; therefore, we expected that T21 could escape from lysosomes. Nevertheless, the efficacy of the NC-T21 was further evaluated and this aspect is discussed in the next sections.

### 3.3. Evaluation of the Nanocapsules Formulation at Different pHs

**Figure 4:**
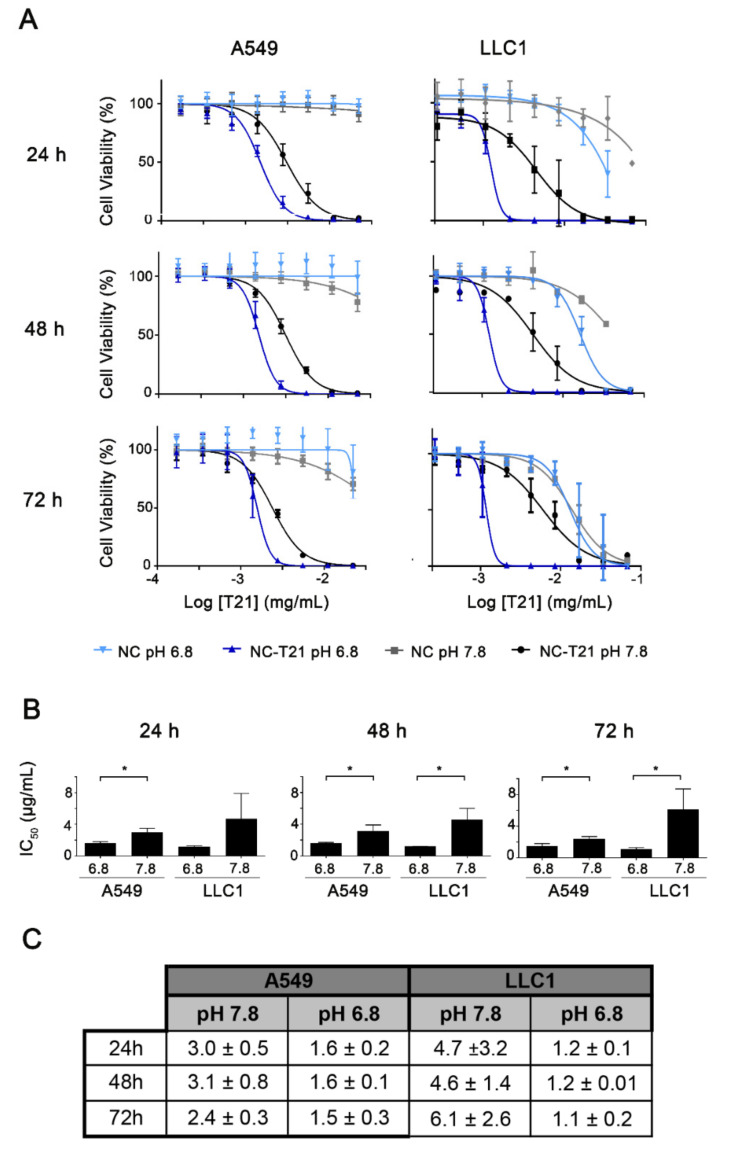
Study of cell viability after NC-T21 treatment dependent on pH. A549 and LLC1 cells were incubated with increasing concentrations of encapsulated T21 (NC-T21) or empty nanocapsules (NC) at pH 6.8 (blue) or 7.8 (black and grey) for 24, 48, and 72 h. (**A**). Cell viability was measured by triplicated MTT assay obtaining cell viability curves where IC_50_ value was extrapolated. (**B**) Comparison of the IC_50_ values. * *p* < 0.05. (**C**) IC_50_ values (µg/mL ± SD), obtained from dose-response curves.

Once probed that the NCs can be accumulated into the cells, we wanted to evaluate whether the amphoteric characteristics of the NCs provide selectivity for acidic environments. For that purpose, cell viability was evaluated by MTT assay at 2 different pHs; 7.8 (representative of normal healthy tissue) and 6.8 (pH that can be found in the tumor microenvironment) in two cell lines (A459 and LLC1). The concentration of the drug needed to reduce cell viability to 50% (IC_50_) is lower at pH 6.8 compared to 7.8 (Figure 4) with higher differences in LLC1 cells. This suggests that the NCs formulation provides selectivity for cells in the acidic tumor microenvironment. Additionally, empty NCs present no significant toxicity for both cell lines at their equimolar IC_50_ of NC-T21, indicating they might be a safe drug carrier. On the other hand, there are no differences in IC_50_ values over time, which indicates that the effect of the T21 is produced during the first 24 h.

**Figure 5:**
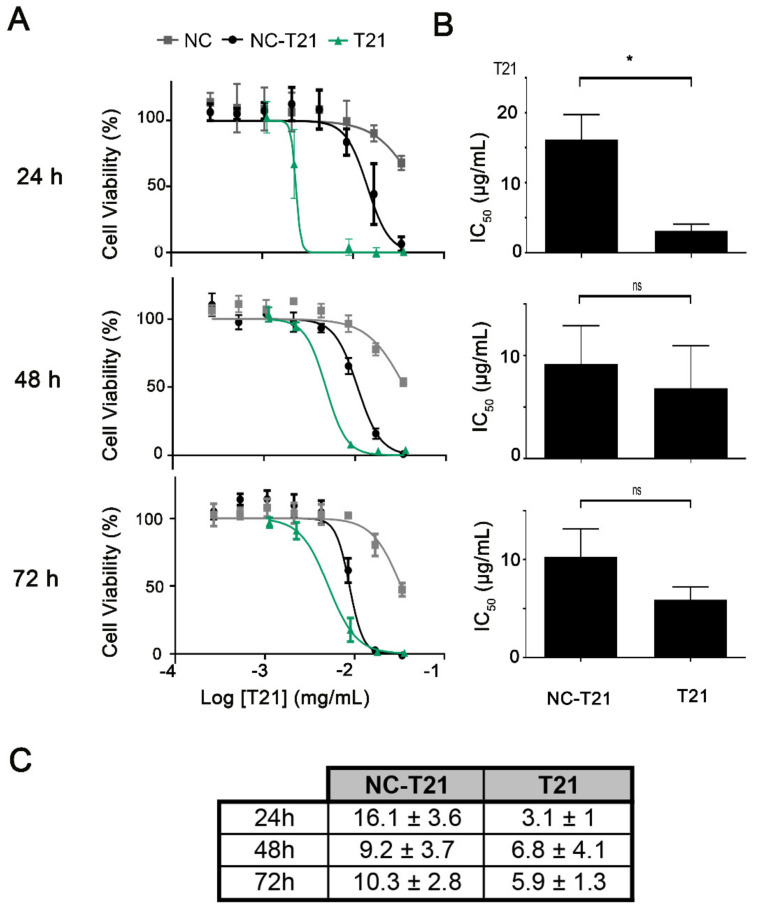
Cell viability in non-tumorigenic cells after treatment with free or encapsulated T21. Non-tumorigenic MCF10A cells were incubated with increasing concentrations of free (T21) and encapsulated T21 (NC-T21) or empty nanocapsules (NC) at pH 7.8 for 24, 48, and 72 h. (**A**) Cell viability was measured by MTT assay obtaining cell viability curves where IC_50_ was extrapolated. (**B**) Comparison of the IC_50_ values. * *p* < 0.05. (**C**) IC_50_ values (µg/mL ± SD), obtained from dose-response curves.

Additionally, the cytotoxic effect of NC-T21 on non-tumorigenic cells was also evaluated and compared to the free drug at pH 7.8 (healthy tissue). Results (Figure 5) showed on one hand, that the IC_50_ of NC-T21 was higher in normal cells than in tumor cells even at pH 7.8 (compared to Figure 4), and on the other hand that the IC_50_ of the free drug (T21) was lower than when it is encapsulated. Altogether, these results suggest the selectivity of these nanocarriers towards the tumor cells, protecting healthy cells from the toxic effect of the drug.

**Figure 6:**
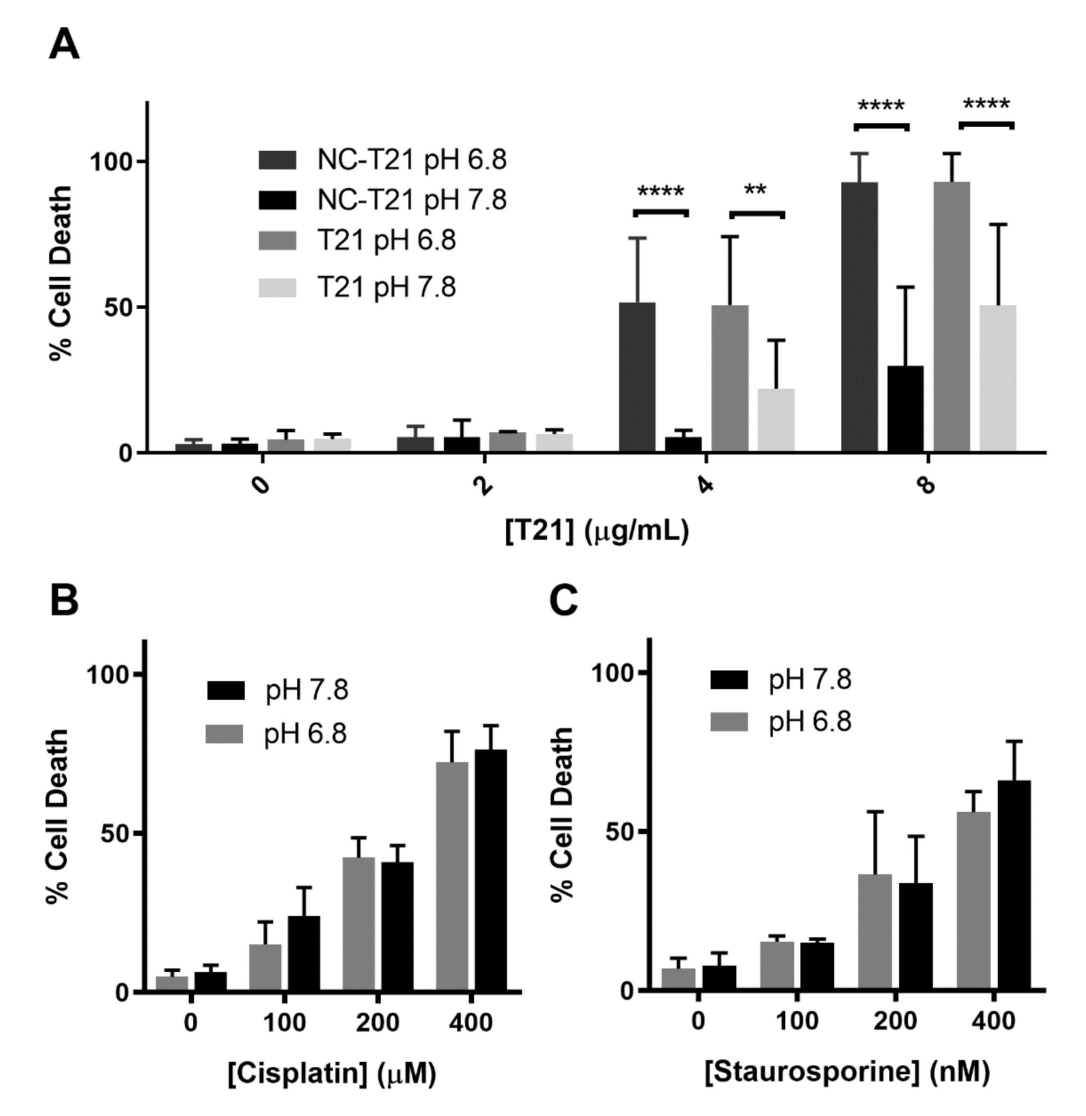
Effect of pH on cell death. A549 cells were incubated with increasing concentrations of (**A**) free (T21) or encapsulated T21 (NC-T21), (**B**) cisplatin, and (**C**) staurosporine at pH 6.8 and 7.8 for 24 h. Dead cells were counted by trypan blue staining, average ± SD of 3 independent experiments is represented. ** *p* > 0.01, **** *p* > 0.0001.

MTT cell viability assay provides a first approach to analyze the effect of a drug on cell lines in different conditions measuring their metabolic activity. Thus, in order to analyze in depth whether NC-T21 is killing cells, cell death was analyzed by trypan blue staining. The results showed that T21 induces more extensive cell death at pH 6.8 than 7.8 (Figure 6A) independently whether it is presented free or encapsulated. It is worth mentioning that the concentration needed to induce cell death is higher than that to affect their metabolic activity (analyzed in Figure 4).

The difference in the predisposition to die at these two pHs was further analyzed to discard sensitization to cell death because of the acidic pH. Therefore, cells were incubated with cisplatin and staurosporine (that present different mechanisms of action, being a DNA-damaging agent and PAN-kinase inhibitor respectively) at pH 6.8 and 7.8, observing no differences on cell death levels (Figure 6B,C). These results suggest that the more acidic pH is not making cells more sensitive to treatment since the toxicity of common drugs, such as cisplatin and staurosporine, is the same at both pHs. Therefore, the observed selectivity of free T21 for acidic pH could be due to the fact that this drug is an anion transporter [27] that acts decreasing intracellular pH (pH_i_) (Figure S6). Thus, it is not surprising that its own cytotoxic effect could be altered by changes in pH_e_.

However, independently of the mechanism of action of the experimental drug, it is worth noticing that at pH 6.8 (representative of tumor microenvironment), both T21 and NC-T21 induce the same percentage of cell death. However, at pH 7.8, the nanoencapsulation of the drug reduces its cytotoxic effect. These results suggest that the nanoencapsulation protects healthy tissue from the cytotoxic effect of the drug; meanwhile, at the tumor site, it exerts the same cytotoxicity than the free drug. On the other hand, the fact that at pH 6.8 both T21 and NC-T21 present same cytotoxic effect also suggests that the efficacy of NC-T21 is not reduced by the internalization mechanism of these NCs (endocytosis) compared to the free drug, which is internalized by passive transport through cell membranes.

**Figure 7:**
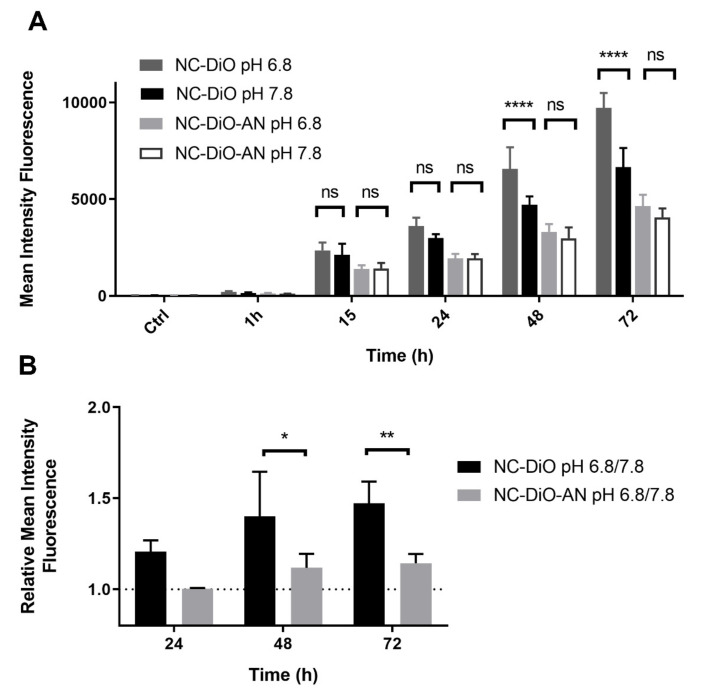
Nanocapsules uptake by flow cytometry. A549 cells were incubated with DiO loaded amphoteric (NC-DiO) or anionic (NC-DiO-AN) NCs at pH 6.8 or pH 7.8 for 1, 15, 24, 48, and 72 h. Fluorescence intensity was measured by flow cytometry. (**A**) Mean intensity fluorescence is represented ± SD of three independent experiments. (**B**) Mean intensity fluorescence ratio at pH 6.8 respect to pH 7.8 is represented. **** *p* > 0.0001; ** *p* > 0.01; * *p* > 0.05 ns: not significant.

After these results, we proceeded with a deeper analysis of cell internalization using a fluorophore dye. It has been shown that change of pH in culture medium does not make cells more sensitive to cell death; however, pH could affect general endocytic pathways. In order to discern the effect of pH in the endocytic process, A549 cells were incubated with amphoteric (NC-DiO) or anionic (NC-DiO-AN) NCs loaded with the fluorophore DiO at pH 6.8 and 7.8. The intensity fluorescence was measured by flow cytometry (Figure 7). According to the results, the interaction of NCs with cells is time-dependent. When cells are incubated with amphoteric NCs, which present positive-charged surface at acidic pHs and neutral to negative at basic pH, medium at pH 6.8 favors NCs internalization compared to pH 7.8. Whereas anionic NCs, which present a negatively charged surface at both pHs, are internalized similarly at pH 6.8 and 7.8. These results support the hypothesis that the amphoteric properties of the NCs provides selectivity for their internalization at acidic pHs, since positively charged nanocarriers are taken by cells more efficiently than those neutral or negatively charged [28].

Altogether, these results support that the NCs of study are internalized better at pH 6.8 than 7.8, resulting in a major cytotoxic effect at slightly acidic pH, the conditions found in tumor microenvironment.

### 3.4. Tumor-Accumulation and Whole-Body Biodistribution of the Nanocapsules

**Figure 8:**
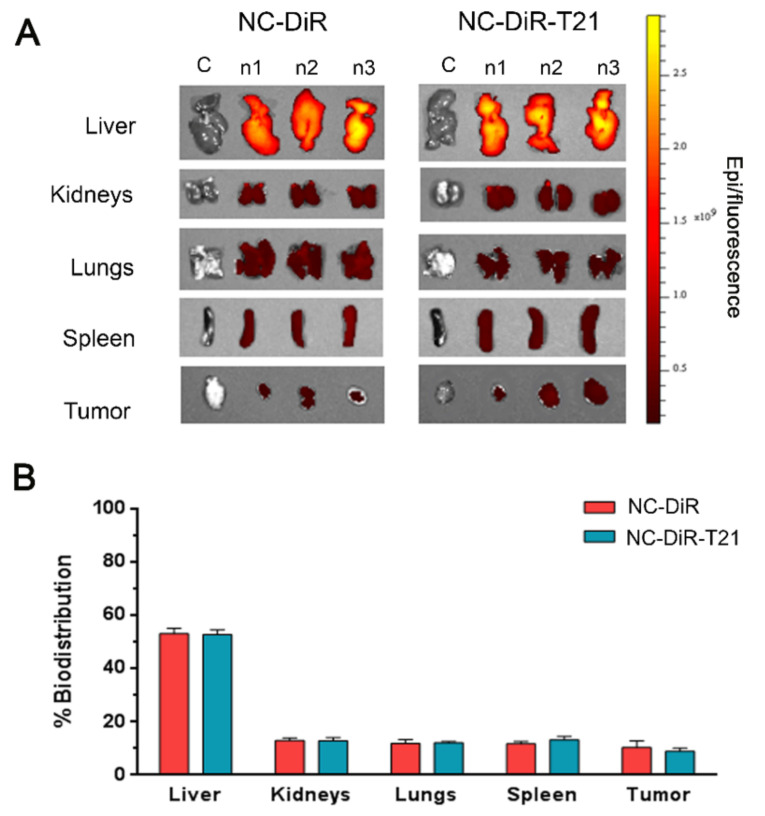
Nanocapsules tissue biodistribution. Mice were administered i.v. with NC-DiR or NC-DiR-T21.Nanoparticle tissue accumulations were determined by ex vivo DiR fluorescence imaging monitoring 72 h post-injection. (**A**) Representative images of the different organs and tumors analyzed. (**B**) Percentage of biodistribution among the indicated tissues after injecting NC-DiR or NC-DiR-T21 nanoparticles. Graph represents the average of total REU/area/tissue ± SD.

Once we probed that these nanocarriers present preference to enter cells in slightly acidic conditions, we wanted to analyze their accumulation in tumors, where the microenvironment is more acidic than in the majority of healthy tissues. Thus, NCs loaded with the fluorophore DiR (NC-DiR) or NCs loaded with the fluorophore DiR and T21 (NC-DiR-T21) were administered to mice bearing subcutaneous tumors. A unique i.v. administration dose of NC-DiR and NC-DiR-T21 at 0.4 mg equivalent DiR/kg was well tolerated and adverse side-effects were not observed up to 72 h post administration. Ex vivo results showed that NC-DiR and NC-DiR-T21 fluorescence was detected mainly in the liver (53% of biodistribution) (Figure 8) as most nanoparticles [29]. NCs were accumulated into tumors, the tumor uptakes of the NC-DiR and NC-DiR-T21 being similar (9–10% of biodistribution). Spleen, lungs, and kidneys showed comparable percentage of nanocapsule accumulation (12–13%). Both nanoparticles showed similar accumulation levels in all the tissues, indicating that the presence of T21 does not modify the biodistribution of nanocapsules. Thus, the nanocapsules protect the drug and their characteristics are responsible for their biodistribution.

### 3.5. Toxicity Study In Vivo

**Figure 9:**
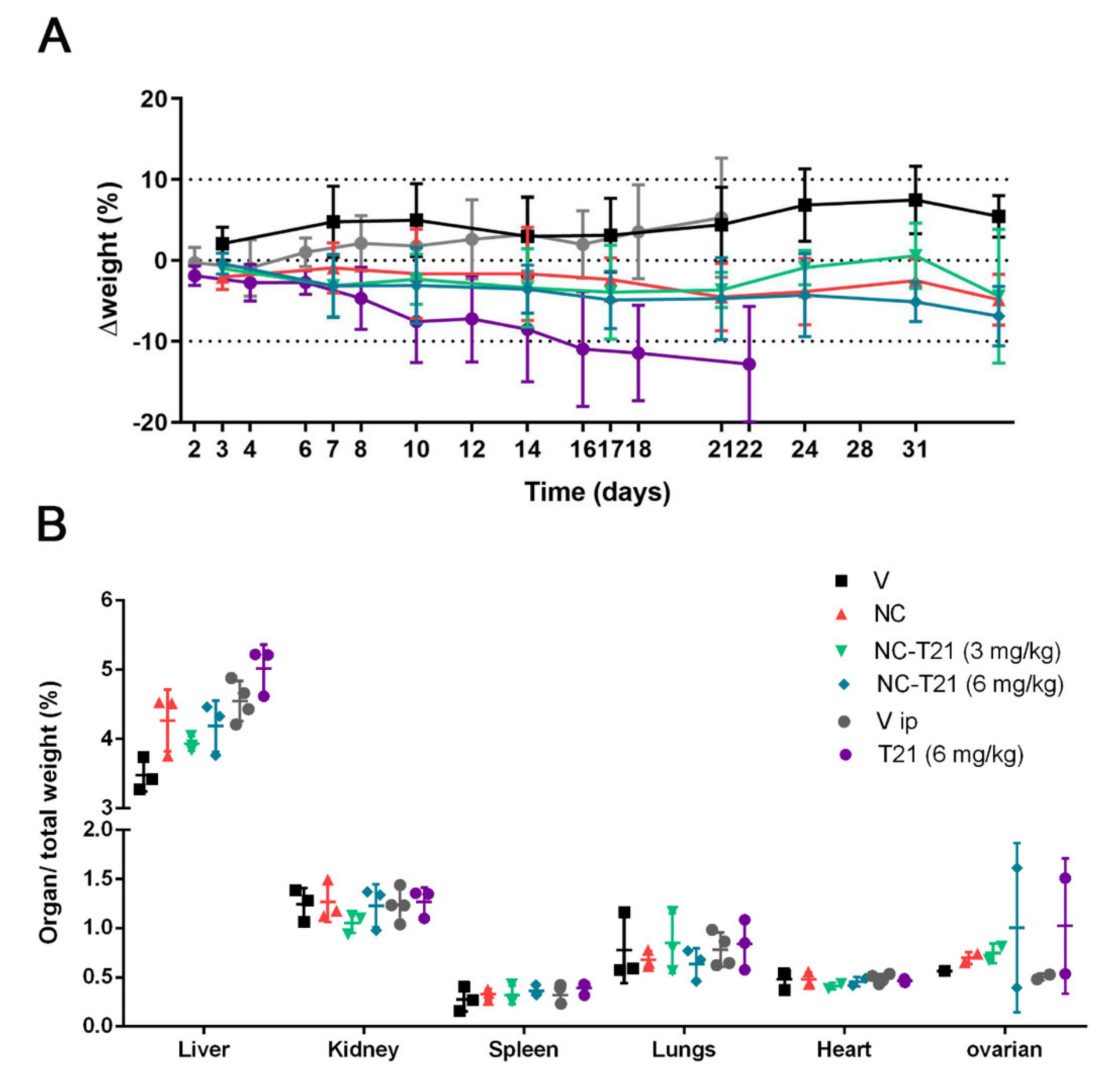
Effect of treatment in mouse growth and organ weight. Mice were administered i.v. with 6 mg/kg or 3 mg/kg of nanoencapsulated T21 (NC-T21), PBS (V), empty nanocapsules without T21 (NC), 6 mg/kg of free T21 (T21) administered i.p. or PBS 7.5% DMSO and 0.8% Tween (Vip). The mice received 10 i.v. doses in a twice a week regimen or every other day for i.p administration. Mouse weight was monitored during treatment; (**A**) Percentage of weight variation during treatment and (**B**) relative organs weight are represented.

In order to evaluate the safety profile of T21 versus NC-T21, mice were administered with 6 mg/kg or 3 mg/kg of NC-T21, with empty nanocapsules (NC) or with the corresponding vehicle, which is PBS (V). Free T21 administered i.v. cause mice death, hence its administration had to be done using a different administration route. This is why, another group was treated with 6 mg/kg of free T21 (T21) or with PBS 7.5% DMSO and 0.8% Tween (Vip) intraperitoneally (i.p.), (Figure 9). The results previously published showed reduction of tumor growth when mice inoculated with DMS53 cells (human lung carcinoma) were treated with 6 mg/kg T21 administered every other day, total dose 60 mg/kg [17]. Thus, in order to mimic this experiment to use it as a control, the mice were treated with T21 i.p using the same dose schedule; however, nanocapsule formulation was administered i.v. and the mice were treated only twice a week.

Mice treated with free T21 showed loss of weight between 10–20% of their initial weight (Figure 9A). However, the groups of mice treated with NC or NC-T21 maintained their weight constant, or it slightly decreased, always being less than 10%. These results suggest a reduction in T21 toxicity when encapsulated. On the other hand, the weight of the organs was not significantly different between groups, except one female reproductive organ in both groups treated with T21, which, due to the limited number of samples, was not possible to discern whether this difference was significant or due to a variance that could depend on the reproductive cycle stage.

These preliminary results seem to indicate that treatment with T21 presents toxicity at an effective dose, which is reduced by its encapsulation, improving T21 in vivo tolerability and allowing its administration intravenously. To confirm this hypothesis, more extensive analysis using the same dose schedule was performed in order to compare both administration routes, analyzing toxicity and efficacy in the same model.

### 3.6. Efficacy Study In Vivo

To analyze the therapeutic effects of NC-T21, an orthotropic lung cancer mice model that resembles human malignancy was used. These mice present an oncogenic mutation in *Kras* (gly → asp codon 12; *Kras^G12D^*) and an LSL cassette with transcriptional and translational stop elements flanked by LoxP sites into the first intron of the *Kras* gene that prevents the expression of the mutant allele until the stop elements are removed by the activity of Cre recombinase. Lung tumors are initiated with the inhalation of viruses expressing Cre that activates the oncogenic *Kras^G12D^* allele [25].

Mice with lung tumors were randomized into four groups and treated twice a week, with NC-T21, PBS, or NC, i.v. The fourth group was treated with T21 administered intraperitoneally (i.p.). All mice were treated twice a week with 6 mg/kg of T21 or the equivalent of empty NCs for 35 days.

**Figure 10:**
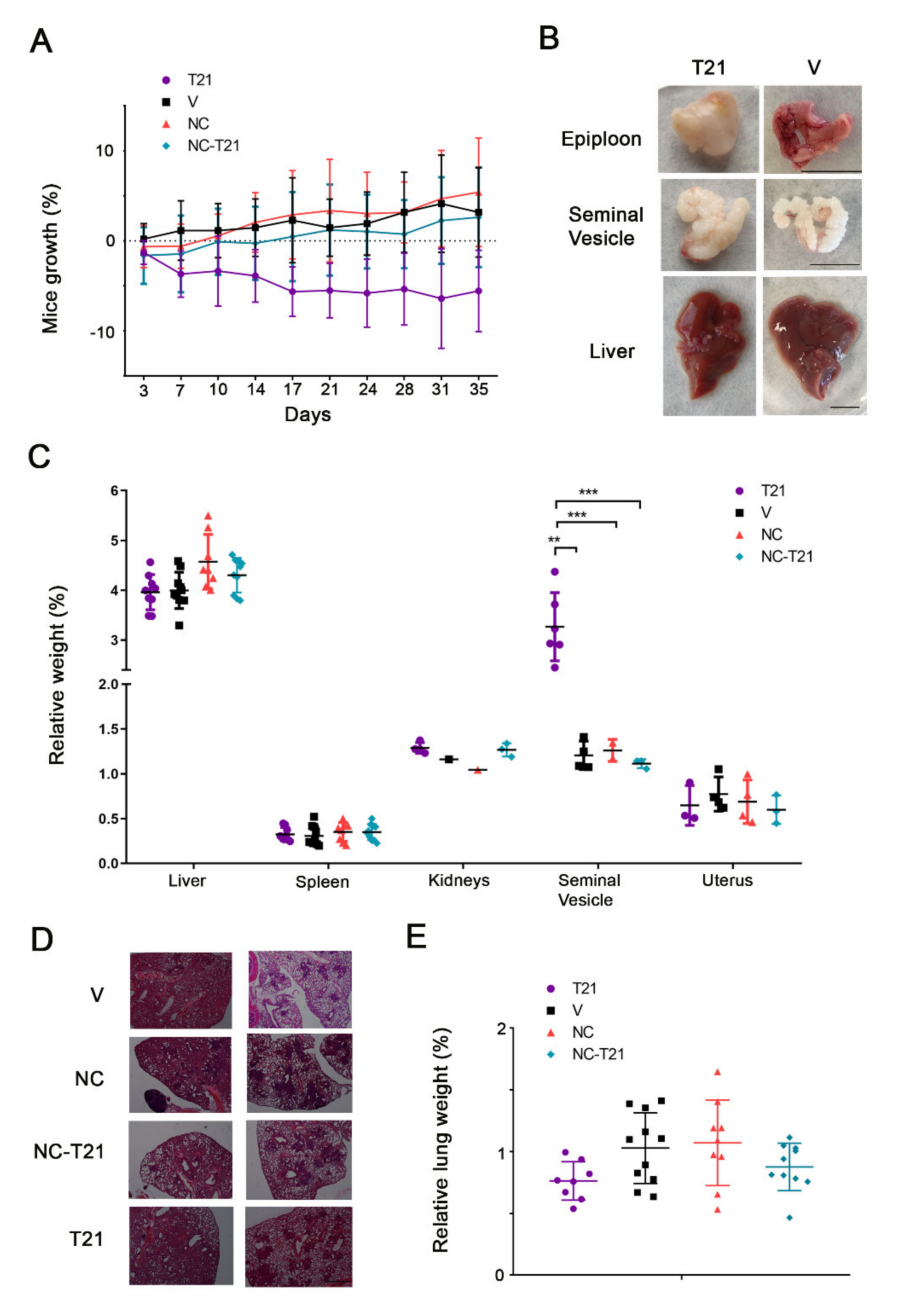
Analysis of the tolerability and therapeutic effect of NC-T21 treatment. Lung tumors were induced in mutant mice by virus inhalation, as described in Materials and Methods. 12 weeks after, mice were treated twice a week, with 6 mg/kg of nanoencapsulated T21 (NC-T21); with PBS (V), with empty nanocapsules without T21 (NC) or with free T21 (T21). (**A**) Mice weight was monitored during treatment. (**B**) Macroscopic differences in epiploon, seminal vesicle, and liver were observed in mice treated with T21. Scale bar 1 cm (**C**) Organs and (**E**) Lungs were weighed at final point and relativized to mice total weight. (**D**) Pictures of hematoxylin/eosin staining of lung tumors were taken. Scale bar 0.5 mm. ** *p* > 0.01, *** *p* > 0.001.

Due to the change of administration schedule for free T21, first we evaluated the tolerability of the drug. Mice in the control group or those treated with NC or NC-T21 showed no differences in their growth or the size of their organs (Figure 10A–C). Nevertheless, despite the fact that the treatment schedule was reduced to twice a week, mice treated with free T21 still lost weight during treatment (Figure 10A). However, some of these mice started to recover weight at day 22 of treatment, which was due to the emergence of a huge mass in the epiploon, the internal fat located in the peritoneum (Figure 10B) that was tougher, bigger, and yellowish. The relative weight of the analyzed organs showed no differences among groups, except the seminal vesicle of mice treated with T21 that showed an increase in size and toughness. On the other hand, in the livers of mice treated with T21, despite the fact that there were no differences in their relative weight, presented a macroscopic unusual morphology, according to the shape of their lobes—more rounded and thicker (Figure 10B). Additionally, it should be noted that the epiploon of the mice treated with T21 was yellowish, which seems to indicate that T21 may be accumulated in fat tissues of the peritoneum area (area of injection), increasing its toxicity when the treatment is prolonged, even though the total dose of T21 was the same as that used in Figure 9. However, none of these effects were observed in the groups treated with NC-T21, NC, or vehicle. Altogether, these results indicate that T21 results toxic and its toxicity is reduced when the drug is encapsulated, which corroborates previous preliminary results.

It should be mentioned that this tumor model presents different sites of tumor initiation, since tumors start growing by virus inhalation in the whole lung. Thus, there are multiple sites of tumor initiation and we can find several tumor progression stages in the same lung [25] (Figure 10D). Due to this characteristic, we could not measure the tumor area or the progression of the disease easily. Therefore, in order to evaluate the extension of the tumor, lungs were weighed (Figure 10E) and results showed a reduction of tumor mass in those mice treated with T21 or NC-T21, which seems to indicate that the NCs formulation allows drug delivery in the tumor site, maintaining its activity.

To date, different type of molecules, mainly antibodies, have been used as bioligands for active target therapy to improve important aspects such as selective cellular internalization, minimizing undesired secondary effects [30]. However, nanocapsules conjugated to proteins modify their antibody 3D structure and the new derivatives are more detectable by RES, being a relatively large and a new not-natural entity, compared to natural peptides or proteins alone, or well-designed hydrophilic capsules separately [31]. Despite the fact that the use of fragments of antibodies has recently opened new possibilities to improve theses bioligand nanoparticles [32], trying to avoid its immunogenicity, this type of targeting is still in preliminary studies [33]. The cost of production of these antibodies and fragments is still a limitation for its further use. Our multi-smart nanocapsules described in this article do not contain proteins or peptides on their surface. They are designed to be neutral and hydrophilic when circulating in the bloodstream to go unnoticed by the RES, and only become cationic in the tumor microenvironment, promoting its accumulation and penetration in dysregulated cancer cells using a characteristic common to all types of tumors, the acidic tumor microenvironment. They are easy to fabricate, versatile to be modified, and adapt to different targeting applications.

Targeting tumors based on their acidic microenvironment is one therapeutic approach to improve the selectivity of cancer treatment that has been studied lately in nanomedicine, not only to design drug delivery systems but also in other therapeutic approaches such as photodynamic [34] or combination [35] therapies. These studies use different methods to increase the concentration of the therapeutic agent in the tumor site; one is targeting tumors based on the pH. For instance, a pH (low) insertion peptide (pHLIP) bonded to the nanoparticle can be used to direct nanoparticles to tumors [34]. Other studies use the difference on the pH to increase the drug release in the tumor microenvironment where the nanocapsules are disintegrated at acidic pH [15,35,36].

We have developed a drug delivery system that facilitates its cellular uptake at acidic pH, where its surface charge becomes cationic. It has been widely studied that positively-charged nanoparticles interact more with the cell membranes, increasing their uptake compared to neutral or negative charged ones [28,37,38]. In our system, higher cellular uptake is accompanied by higher cellular death extension at slightly acidic pH compared to physiological pH. Once the nanoparticles are inside the cells, the drug is released by high concentration of GSH, as shown in a previous work [24]. This fact protects healthy organs from the action of the drug, reducing its secondary effects, as shown in our in vivo experiments. Additionally, the cytotoxic activity of the drug is maintained in tumors, showing similar efficacy as the free drug. Our results open a new opportunity to develop safer drug delivery platforms and allow the administration of hydrophobic drugs intravenously.

## 4. Conclusions

We studied both in vitro and in vivo multi-sensitive nanocapsules loaded with the antitumor drug T21. Our results indicate that these amphoteric nanoencapsules are internalized better at slightly acidic pH, found in the tumor microenvironment, compared to that found in healthy tissue. This higher internalization is accompanied by a higher percentage of cell death. Additionally, a biodistribution assay demonstrated that NCs are able to reach the tumor. More importantly, our results indicate that the nanoencapsuation of T21 diminishes its systemic toxicity, while maintaining its desired anti-tumor cytotoxic effect. As an added value of this work, and in contrast to other current drug delivery nanosystems, these nanocarriers are prepared by industrially scalable methods using bulk starting materials, and can be applicable to several commonly used hydrophobic drugs, like many current chemotherapeutics. Therefore, this approach could represent a powerful tool to improve the performance and safety of a wide range of cytotoxic molecules currently used in cancer treatment.

## Supplementary Materials

The following are available online at https://www.mdpi.com/article/10.3390/biomedicines9050508/s1, Figure S1: Molecular structure of antitumor drug T21, Figure S2: IR Spectra of PI/P2 synthesis, Figure S3: IR Spectra of the encapsulation reaction, Figure S4: Calibration plot for T21 and DL, EE parameters, Figure S5: pH measurement, Figure S6: Variation of extracellular pH after T21 treatment. Table S1: Amounts of reagents used to prepare P2, Table S2. Amounts of reagents used to prepare NC-DiO-AN, Table S3. Amounts of reagents used to prepare NC-DiR, Table S4. Amounts of reagents used to prepare NC-T21, Table S5. Amounts of reagents used to prepare NC-DiR-T21, Table S6. Amounts of reagents used to prepare NC.
