# Supplementary material for: Multi-Smart and Scalable Bioligands-Free Nanomedical Platform for Intratumorally Targeted Tambjamine Delivery, a Difficult to Administrate Highly Cytotoxic Drug
Source: Biomedicines. 2021 May 4;9(5):508. doi: 10.3390/biomedicines9050508 (PMC8147975; doi:10.3390/biomedicines9050508)
Supplement: Supplementary file 1 [file biomedicines-09-00508-s001.zip › biomedicines-1176129-supplementary.pdf]

biomedicines

Supplementary information

Multi-smart and scalable bioligands-free nanomedical platform for intratumorally targeted Tambjamine Delivery, a difficult to administrate highly cytotoxic Drug.

1. Materials

1.1. Building blocks and crosslinkers: Isophorone diisocyanate IPDI was purchased from Quimidroga (Barcelona, Spain), YMER N-120 was supplied by Perstorp (Perstorp, Sweden) and Genamin TAP 100D was provided by Clariant (Barcelona, Spain). Jeffcat DPA and DEDS, DETA and L-lysine hydrochloride were purchased from Sigma Aldrich (St Louis, USA).

1.2. Encapsulated molecules: DiO and DiR were purchased from Thermo Fisher Scientific (Barcelona, Spain). NALA was purchased from Sigma Aldrich. Compound T21 was synthesized according to published procedures.[1]

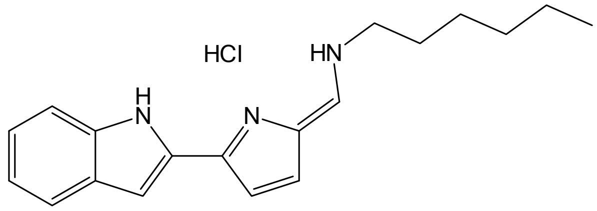

Figure S1. Molecular structure of antitumor drug T21

1.3. Solvents and auxiliary solutions: Milli-Q water was obtained from a Merck Millipore purification system (Madrid, Spain) and PBS, HCl 37% (by wt) and NaOH in pellets were purchased from Merck (Madrid, Spain).

2. Analytical techniques

The present work, including most of the characterization techniques, has been developed between Ecopol Tech's facilities and the Organic Chemistry section from the Inorganic and Organic Department of University of Barcelona. TEM and flow cytometers were used from Scientific and Technological Centers of University of Barcelona.

2.1. Infrared spectroscopy (IR): IR spectra were performed in a Smart ATR (Nicolet iS10, Thermo Scientific, Raleigh, USA) using a transmittance mode (32 scans) and OMNIC software. For the monitoring of solvent-based samples, one drop was deposited onto the diamond crystal and the solvent was left to dry by evaporation. IR spectra were recorded from a dry film of the sample for the reaction control after emulsification.

2.2. pH measurements: The pH of the emulsion was determined right after the crosslinker was added and at different time intervals until the last polyaddition reaction was complete. All the determinations were carried out in a pH-meter HI 2211 pH/ORP-Meter (HANNA Instruments, Eibar, Spain) equipped with a pH electrode Crison 5029 (Crison Instruments, Barcelona, Spain) and a temperature probe.

2.3. Dynamic light scattering (DLS): The size distribution of the NCs was analyzed on a Zetasizer Nano-ZS90 (Malvern, Worcestershire, UK) in Milli-Q water at 25 °C at a concentration of 1 mg/mL.

2.4. Zeta potential (Z-pot): The Z-pot of the NCs was analyzed on a Zetasizer Nano-ZS90 (Malvern, Worcestershire, UK) in Milli-Q water at 25 °C at a concentration of 5 mg/mL, measured at different pH values.

2.5. Transmission electron microscopy (TEM): The morphology of NCs and the in vitro degradation in presence of reduced GSH were studied in a Jeol JEM 1010 (Jeol, Peabody, USA). A 200-mesh copper grid coated with 0.75% FORMVAR was deposited on 6 μL of an aqueous NCs suspension at 10 mg/mL for 1 min. The excess of sample was removed by contact with a drop of Milli-Q water for 1 min and the grid was deposited on a drop of uranyl acetate (2% aqueous solution) in Milli-Q water for 1 min. The excess of uranyl acetate was removed, and the grid was air-dried for at least 3 h prior to measurement.

2.6. Ultraviolet/visible spectroscopy (UV/Vis): The drug loading (DL) of the NCs was determined by UV-Vis measurements performed in a Cary 500 Scan UV-Vis-NIR spectrophotometer (Varian, Palo Alto, USA). First, a calibration curve was developed by preparing a range of standard solutions containing the drug at different concentrations and analyzing their UV profile and maximum absorbance (see figure S4). Then, Encapsulation Efficiency EE (%) and Drug Loading DL (%) were calculated from the following equations:

$$\% EE = \frac{amount\ of\ drug\ incorporated\ in\ the\ nanocapsule}{total\ amount\ of\ drug\ added\ in\ the\ aqueous\ dispersion} * 100$$

$$\% DL = \frac{amount\ of\ drug\ incorporated\ in\ the\ nanocapsule}{total\ amount\ of\ lyophilized\ nanocapsules} * 100$$

To determine the amount of drug incorporated in the NCs, a desired amount of lyophilized NCs (previously dialyzed) was dissolved in an exact volume of solvent and the measurement was calculated from the calibration curve. Unloaded NCs were used as the reference. All measurements were recorded at the desired wavelength range depending on the molecule under study and assayed in triplicates.

2.7. Lyophilization and reconstitution: Lyophilization was carried out with purified aqueous samples in a Christ Alpha 2-4 LDplus freeze dryer (Martin Christ, Osterode am Harz, Germany). Previously dialyzed samples were lyophilized and redispersed in the desired solvent depending on the characterization method.

2.8. Solids concentration: NCs concentration in the aqueous dispersion was determined by triplicate using an OHAUS MB25 moisture analyzer (Ohaus, Greifensee, Switzerland), with a temperature range from 23 °C to 160 °C applying 5 °C increments.

2.9. Dialysis purification: The NCs were dialyzed against distilled water for 24 h for chemical characterization and against Milli-Q water for 72 h for biological assays using a Spectra/Por molecular porous membrane tubing with a 12–14 kDa molecular MWCO (Spectrum Laboratories, Rancho Dominguez, USA). For the *in vivo* experiments, the NCs were dialyzed against PBS 0.01 M (0.138 M NaCl; 0.0027 M KCl; pH 7.4 at 25 °C) instead.

3. Synthetic procedures

3.1. Amphiphilic cationic polymer (P1)

2,2′-Dihydroxyethyl disulfide (DEDS) (381.4 mg, 4.94 meq), YMER N-120 (4.54 g, 8.74 meq) and *N*-(3-dimethylaminopropyl)-*N,N′*-diisopropanolamine (Jeffcat DPA) (381.1 mg, 3.49 meq) were added into a three-necked round-bottom flask equipped with mechanical stirring at room temperature (rt) and purged with N_2. When the mixture was homogeneous, isophorone diisocyanate IPDI (3.24 g, 29.15 meq) was added into the reaction vessel under gentle mechanical stirring. The polyaddition reaction was kept under these conditions until the NCO stretching band intensity did not change, monitored by IR spectroscopy. At this point, dry THF (10 mL) was added into the reaction mixture in order to fluidify the polymer. In parallel, 1,3-diamino-*N*-octadecylpropane (Genamin TAP 100D) (2.54 g, 15.03 meq) was dissolved with dry THF (10 mL) into another 100 mL three-necked round-bottom flask, which had previously been purged with N_2 and precooled to 4 °C. The former reaction mixture was added dropwise onto the latter under smooth mechanical stirring. The reaction was monitored by IR until the NCO stretching band intensity had completely disappeared.

3.2. Amphiphilic polymer (P2)

The procedure was based on the synthesis of P1 (see section 3.1) with slight variations, detailed in **Table S1**.

Table S1. Amounts of reagents used to prepare **P2**

Substance	Weight	Equivalents
**DEDS**	370.6 mg	4.81 meq
**YMER N-120**	4.42 g	8.51 meq
**Jeffcat DPA**	0 g	0 meq
**IPDI**	2.34 g	23.98 meq
**Genamin TAP 100D**	3.16 g	13.86 meq

3.3. DiO-loaded amphoteric NCs (NC-DiO)

IPDI (30.9 mg, 0.28 meq) was added into a three-necked round-bottom flask equipped with mechanical stirring, precooled at 4 °C, purged with N_2 and protected from light. In parallel, the product to be encapsulated, DiO in this case (4.0 mg, 4.5 eq), polymer P1 (242.8 mg of P1, 0.069 meq) and dry THF (1.5 mL) were mixed in a vial, added into the flask and left to homogenize for 30 min at 150 rpm, protected from light. At this point, an alkaline aqueous solution of L-lysine was prepared by dissolving 1.19 g L-lysine in 9.33 g of Milli-Q water and adjusting pH to 11.0 with alkaline NaOH solutions at 3 M and 1 M (total L-lysine concentration 8.2% by wt). This solution (5.8 mg of L-lysine, 0.070 meq) was added and the polyaddition reaction was checked after 45 min by IR. Then, the organic phase was emulsified at 300 rpm with cold Milli-Q water (15.03 g) and finally an aqueous solution of diethylenetriamine (DETA) (4.2 mg of DETA, 0.12 meq) was added in order to generate crosslinked NCs from the nanodroplets present in the emulsion. The stirring was reduced to 100 rpm and the ice bath was removed to let the reaction reach rt. This polyaddition reaction was monitored by IR and pH measurements. Once the NCs were formed, THF was removed from the reactor at 40 °C under reduced pressure and the pH was adjusted to 7.0 using diluted aqueous HCl.

3.4. DiO-loaded anionic NCs (NC-DiO-AN)

The procedure was based on the DiO-loaded amphoteric NCs (see section 3.3) with slight variations, detailed in **Table S2**.

Table S2. Amounts of reagents used to prepare **NC-DiO-AN**

Substance	Amount	Equivalents
**IPDI**	22.1 mg	0.19 meq
**Fluorophore (DiO)**	2.9 mg	–
**Polymer (P2)**	212.2 mg	0.06 meq
**Dry THF**	1.5 mL	–
**L-lysine**	8.1 mg	0.09 meq
**Milli-Q water**	15.03 g	–
**DETA**	3.4 mg	0.09 meq

3.5. DiR-loaded amphoteric NCs (NC-DiR)

The procedure was based on the DiO-loaded amphoteric NCs (see section 3.3) with slight variations, detailed in **Table S3**.

Table S13. Amounts of reagents used to prepare **NC-DiR**

Substance	Amount	Equivalents
**IPDI**	39.3 mg	0.35 meq
**Fluorophore (DiR)**	3.8 mg	–
**Polymer (P1)**	328.4 mg	0.063 meq
**Dry THF**	1.2 mL	–
**L-lysine**	11.55 mg	0.14 meq
**Milli-Q water**	10.01 g	–
**DETA**	4.2 mg	0.12 meq

3.6. Tmbj21-loaded amphoteric NCs (NC-T21)

The procedure was based on the DiO-loaded amphoteric NCs (see section 3.3) with the exception that NALA was mixed with the drug and the polymer. The exact amounts are detailed in **Table S4**.

Table S24. Amounts of reagents used to prepare **NC-T21**

Substance	Amount	Equivalents
**IPDI**	73.9 mg	0.46 meq

**T21**	99.6 mg	–
**NALA**	56.4 mg	–
**Polymer (P1)**	745.5 mg	0.16 meq
**Dry THF**	1.6 mL	–
**L-lysine**	21.4 mg	0.25 meq
**Milli-Q water**	14.06 g	–
**DETA**	6.7 mg	0.19 meq

3.7. T21 and DiR-loaded amphoteric NCs (NC-DiR-T21)

The procedure was based on the DiO-loaded amphoteric NCs (see section 3.3) with the exception that DiR, T21 and NALA were mixed together with the polymer. The exact amounts are detailed in **Table S5**.

Table S5. Amounts of reagents used to prepare **NC-DiR-T21**

Substance	Amount	Equivalents
**IPDI**	27.2 mg	0.19 meq
**Fluorophore (DiR)**	8.6 mg	–
**T21**	35.5 mg	–
**NALA**	21.7 mg	–
**Polymer (P1)**	264.5 mg	0.051 meq
**Dry THF**	1.2 mL	–
**L-lysine**	7.2 mg	0.1 meq
**Milli-Q water**	5.05 g	–
**DETA**	3.2 mg	0.1 meq

3.8. Empty NCs (NC)

The procedure was based on the DiO-loaded amphoteric NCs (see section 3.3) with the exception that no fluorophore was added with the polymer and the flask was not protected from light. The exact amounts are detailed in Error! Reference source not found.**S6**.

Table S6. Amounts of reagents used to prepare **NC**

Substance	Amount	Equivalents
**IPDI**	70.5 mg	0.49 meq

**Fluorophore (DiO)**	0 mg	–
**NALA**	59.6 mg	–
**Polymer (P1)**	736.3 mg	0.14 meq
**Dry THF**	1.2 mL	–
**L-lysine**	20.9 mg	0.25 meq
**Milli-Q water**	13.50 g	–
**DETA**	7.9 mg	0.23 meq

4. Additional spectra

4.1. Infrared Spectra

The polymerization reaction could easily be controlled by IR spectroscopy using the procedure detailed in section 2.1. given that NCO has a very clear and characteristic stretching band at 2280–2230 cm^{-1}. The polymerization reaction (P1/P2) is shown in **Figure S2**.

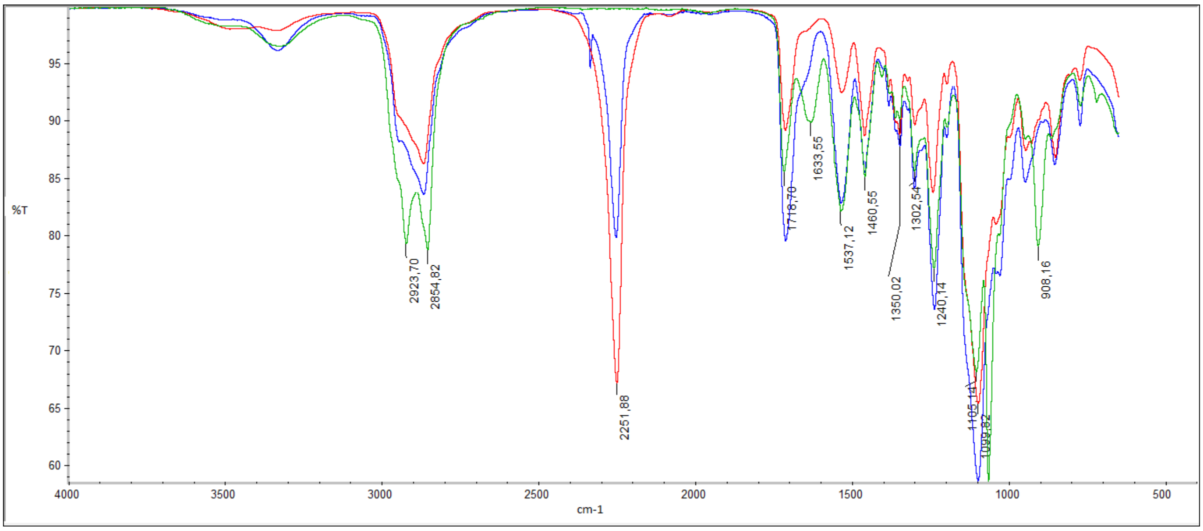

Figure S2. IR Spectra of PI/P2 synthesis. IR spectra of the polymers (P1 or P2) indicated a successful polyaddition reaction between the diols, the diamine and the diisocyanate, in both steps of the polymer synthesis. The red line corresponds to the first sample recorded, at the start of the reaction. At that time, the NCO asymmetric stretching band at 2252 cm^{-1} was very sharp and intense. At the end of the first step, involving the reaction between the diols and the diisocyanate (blue line), the intensity of the NCO stretching band decreased significantly. Meanwhile, the intensities of the CO stretching band at 1719 cm^{-1}, the CN stretching band at 1537 cm^{-1}, the NCOO/COC asymmetric stretching band at 1240 cm^{-1} increased. Overall, the IR spectra performed during the first step of the synthesis confirmed polyurethane bond formation along with NCO consumption. Once the diamine was added during the second step of the polymer synthesis (green line), the NCO stretching band at 2252 cm^{-1} disappeared instantaneously, which was explained by the high reactivity of the amines. Simultaneously, other characteristic bands appeared or changed, such as a new stretching band at 1634 cm^{-1}, which was associated to the carbonyl of urea bonds and a new wagging band at 908 cm^{-1} corresponding to the free secondary amine, which also confirmed polyurea formation.

The encapsulation reaction was also controlled by IR spectroscopy as shown in **Figure S3**.

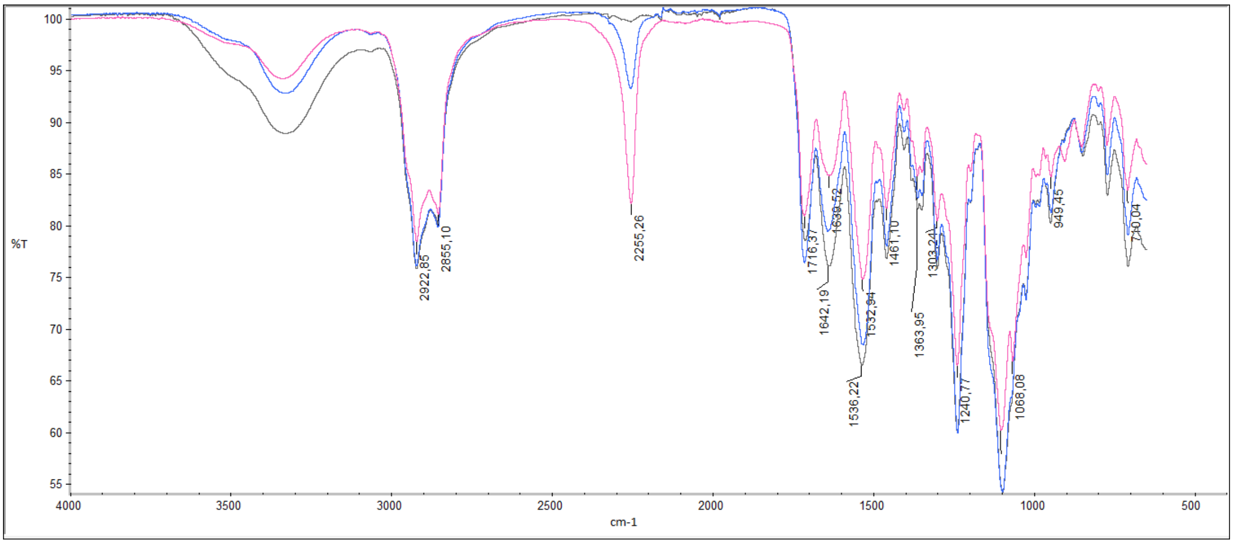

Figure S3. IR Spectra of the encapsulation reaction

IR spectra of the NCs (regardless their loading) indicated also a successful nanocapsule formation. The pink line in the IR spectra represents the sample 30 min after the polymer, together with the drug/dye, was mixed with the diisocyanate. This initial step was the reactivation of the polymer and its conversion to a NCO-reactive entity. Afterwards, L-lysine sodium salt was added (blue line) and reacted with the activated polymer. A decrease on the intensity of the NCO stretching band at 2255 cm^{-1}, concomitantly with an increase of the carbonyl and CN stretching bands, confirmed urea formation (1642 cm^{-1} and 1532 cm^{-1}, respectively). Finally, the triamine was added (grey line) and the NCO stretching band instantaneously disappeared and the urea-associated bands increased their intensity as a result of the rapid reaction between remaining NCO groups and this polyamine.

4.2. *UV/Vis Spectroscopy*

Drug Loading (DL) and Encapsulation Efficiency (EE) were determined using UV/Vis spectroscopy, as detailed previously in section 2.6. The calibration plot and the DL and EE parameters for NC-T21 are shown below in **Figure S4**:

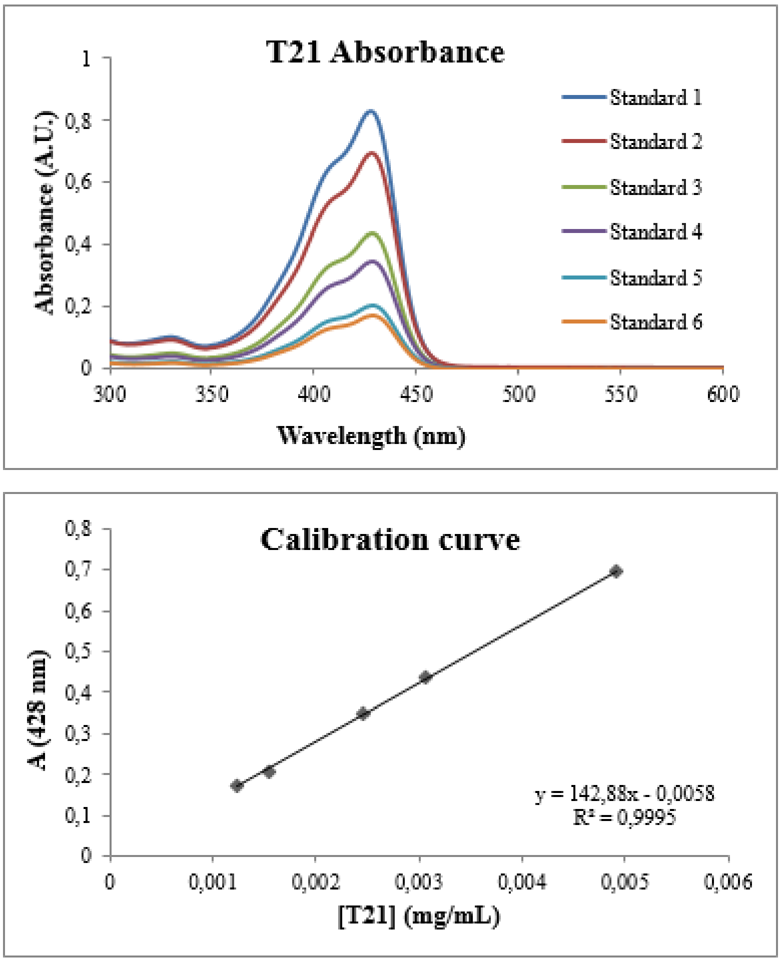

Sample	[T21] (mg/mL)	DL (%)	EE (%)
NC-T21	3.40 ± 0.023	6.66 ± 0.13	51.21 ± 0.97

Figure S4. Calibration plot for T21 and DL, EE parameters

5. Acidic medium characterization

For *in vitro* studies slightly acidic medium was prepared and compared to commercial medium. Commercial DMEM contains 44 mM $NaHCO_3$ which provides a pH between 7.5 - 7.8 after overnight incubation at 5% CO_2. In order to prepare slightly acidic medium, 3 mM $NaHCO_3$ was added to DMEM without $NaHCO_3$ maintaining the pH at 6.8 – 7 as shown in **Figure S5**.

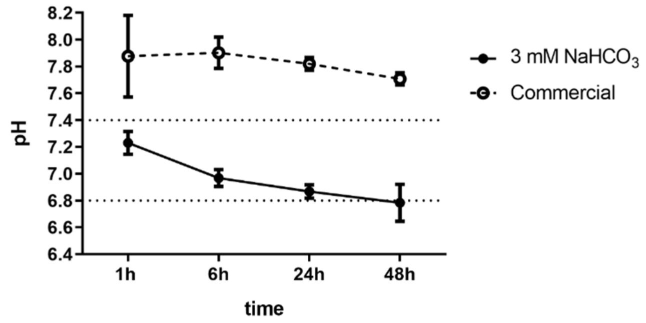

Figure S5 pH measurement. A549 cells were incubated with commercial medium (44 mM HCO_3^-) or medium with 3 mM of HCO_3^- and pH was monitored for 48 h.

6. T21 treatment decreases pH of culture medium.

A549 cells were seeded at $3x10^4$cells/cm^2 in 6 well plate for each treatment or in a p100 for the calibration curve and incubate o/n to allow attachment. Then, cells were treated with 3.6 µg/L of T21 or EH191 (non-anion-transporter) for 1, 4 and 24 h. For calibration, cells were incubated 24 h without treatment. Then treated cells were trypsinized, counted and resuspended in PBS at 10^6c/mL and incubated with 20 µM of SNARF5 (Invitrogen) at 37°C for 30 min. Treated cells were centrifuged, resuspended in PBS at 10^6 c/mL and transferred to cytometer tubes. Cells for calibration were centrifuged and resuspended in PBS at 10^8 c/mL. $5x10^5$ cells (5 µL) were added to each cytometer tube containing 500 µL of high potassium calibration buffer (140 mM KCl, 1 mM $MgCl_2$, 2mM $CaCl_2$, 5 mM D-glucose and 20 mM Bis-tris-propane) at different pHs adjusted with HCl, ranging from 6.2 to 8. Then, cells were incubated with 2 µg/mL of nigericine for 20 minutes at 37°C to allow the equilibration of pH_i to pH_e. The ratio of the emission at 580/620 (ex 488) which is proportional to the pH_i, was calculated by flow cytometry using the cytometer Gallios from Beckman Coulter (Indianapolis, In, USA). **Figure S6** shows that T21 treatment decrease pH_i in one hour; then, it starts to recover to initial values in the next 24h. However, the control EH191 which presents similar structure, but it is not an anion-transporter has no effect on pH_i.

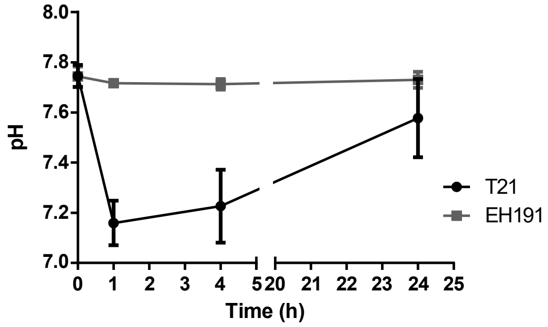

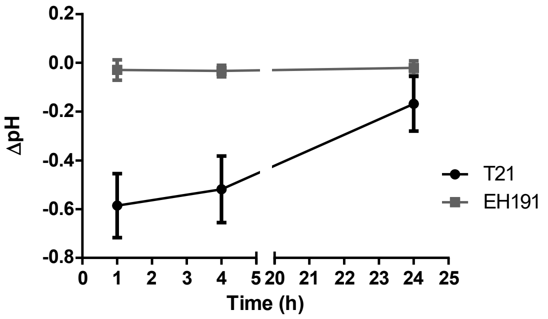

Figure S6: Variation of extracellular pH after T21 treatment. A549 cells were incubated with 3.6 µg/L of T21 (black) or EH191 (grey) for 1, 4 and 24 h. (A and B) pH_i has been measured by flow cytometry using the probe SNARF5. (B) Differences between pH_i respect to control (non-treated cells) were represented.
